# Organic Diets Significantly Lower Children’s Dietary Exposure to Organophosphorus Pesticides

**DOI:** 10.1289/ehp.8418

**Published:** 2005-09-01

**Authors:** Chensheng Lu, Kathryn Toepel, Rene Irish, Richard A. Fenske, Dana B. Barr, Roberto Bravo

**Affiliations:** 1 Department of Environmental and Occupational Health, Rollins School of Public Health, Emory University, Atlanta, Georgia, USA; 2 Department of Environmental and Occupational Health Sciences, University of Washington, Seattle, Washington, USA; 3 National Center for Environmental Health, Centers for Disease Control and Prevention, Atlanta, Georgia, USA

**Keywords:** children’s pesticide exposure, chlorpyrifos, dietary pesticide exposure, malathion, organic diet, organophosphorus pesticides, urinary biomonitoring

## Abstract

We used a novel study design to measure dietary organophosphorus pesticide exposure in a group of 23 elementary school-age children through urinary biomonitoring. We substituted most of children’s conventional diets with organic food items for 5 consecutive days and collected two spot daily urine samples, first-morning and before-bedtime voids, throughout the 15-day study period. We found that the median urinary concentrations of the specific metabolites for malathion and chlorpyrifos decreased to the nondetect levels immediately after the introduction of organic diets and remained nondetectable until the conventional diets were reintroduced. The median concentrations for other organophosphorus pesticide metabolites were also lower in the organic diet consumption days; however, the detection of those metabolites was not frequent enough to show any statistical significance. In conclusion, we were able to demonstrate that an organic diet provides a dramatic and immediate protective effect against exposures to organophosphorus pesticides that are commonly used in agricultural production. We also concluded that these children were most likely exposed to these organophosphorus pesticides exclusively through their diet. To our knowledge, this is the first study to employ a longitudinal design with a dietary intervention to assess children’s exposure to pesticides. It provides new and persuasive evidence of the effectiveness of this intervention.

The National Research Council (NRC) report *Pesticides in the Diets of Infants and Children* (NRC 1993) concluded that dietary intake represents the major source of pesticide exposure for infants and children, and this exposure may account for the increased pesticide-related health risks in children compared with adults. However, direct quantitative assessment of dietary pesticide exposure in children to support this conclusion is no simple task: Several studies (Adgate et al. 2000; Fenske et al. 2002; Gordon et al. 1999; MacIntosh et al. 2001) have analyzed pesticides in representative samples of children’s food, and only two have used biologic monitoring to specifically examine dietary exposures (Curl et al. 2003; MacIntosh et al. 2001). The paucity of exposure data renders the debate over pesticide-related health risks in children controversial (Flower et al. 2004; Garry 2004; Reynolds et al. 2005). Nevertheless, those studies have provided valuable information on dietary pesticide exposure among children and have prompted the needs to improve research methods in order to better assess children’s exposure to pesticides through dietary intake.

The primary objective of this study is to use a novel study design to determine the contribution of daily dietary pesticide intake to the overall pesticide exposure in a group of elementary school-age children using a longitudinal approach. Here we report only results of urinary specific metabolites of organophosphorus (OP) pesticides, a group of insecticides known to cause neurologic effects in animals and humans, for the summer 2003 sampling period. Results of pyrethroid pesticides for the same summer sampling period, as well as results from other sampling periods, will be reported as soon as they become available.

## Materials and Methods

### Subject recruitment.

Twenty-three children 3–11 years of age were recruited from local public elementary and Montessori schools in the suburban Seattle, Washington, area. A letter and a fact sheet describing the study were sent home with children. Families that were interested in participating contacted the research group directly by telephone or e-mail. Schools did not provide any assistance in recruiting subjects. A screening questionnaire was conducted over the telephone to confirm eligibility, which includes children exclusively consuming conventional diets and spending most of their time in one residency, with parents or caregivers willing to provide assistance in collecting specimen samples and other study-related information. Once a subject was enrolled, an in-house appointment was made to go over the study protocol and to obtain written consent from parents and older children, or oral assent from younger children. A questionnaire was also administered during this appointment that asked about household pesticide use to account for other sources of possible pesticide exposure. The University of Washington Human Subject Division approved the use of human subjects in this study.

### Sampling period.

Each child committed to a 15-consecutive-day sampling period, which consisted of three phases. Children consumed their conventional diets during phase 1 (days 1–3) and phase 3 (days 9–15). During phase 2 (days 4–8), organic food items were substituted for most of children’s conventional diet, including fresh fruits and vegetables, juices, processed fruit or vegetables (e.g., salsa), and wheat- or corn-based items (e.g., pasta, cereal, popcorn, or chips) for 5 days. These food items are routinely reported to contain OP pesticides [U.S. Department of Agriculture (USDA) 2005]; we used data from the years 2000–2003. OP pesticides are not regularly detected in meats and dairy products, so these food items were not substituted.

All organic food items were purchased by the research staff from a single grocery store. Parents were asked to request organic foods for their children in phase 2 with the goal of exactly replacing the items the children would have normally eaten as part of their conventional diet. This method ensured that any detectable change in dietary pesticide exposure would be attributable to the organic food rather than a change in the diet. Each child’s daily dietary consumption was recorded by a parent in a food diary throughout the study period. Organic food items, mostly juices and fresh vegetables and fruits, were purchased before and during the study period and analyzed by one of the laboratories contracted by the USDA Pesticide Data Program (PDP) in Yakima, Washington, to confirm that the food items were indeed free of pesticides. No OP or other pesticides were detected in any of the organic food items analyzed.

### Urine sample collection and analysis.

For 15 consecutive days, parents collected urine samples from their child’s first morning voids and the last voids before bedtime. Urine samples were collected daily, refrigerated or maintained on ice before processing in the lab, and then stored at –20°C until pesticide metabolite analysis was performed (Olsson et al. 2004) at the National Center for Environmental Health in the Centers for Disease Control and Prevention in Atlanta, Georgia. Metabolites for selected OP pesticides, pyrethroid insecticides, and herbicides in the urine samples were analyzed; the limits of detection (LODs) for the OP metabolites are listed in Table 1.

### Data management.

Concentrations of OP specific metabolites were reported as three categories, detectable (> LOD), detectable but not quantifiable (< LOD), and nondetectable (ND). For data analysis purposes, reported concentrations for samples with > LOD and < LOD were used, whereas zero was assigned for ND samples. The daily volume-weighted average (DVWA; micrograms per liter) of OP pesticide metabolites was calculated (Equation 1) by averaging the metabolite concentration in the morning sample with that of the previous day’s bedtime sample and then normalizing for the total volume of these two urine samples:


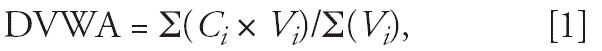


where *C_i_* is the individual urinary concentration (micrograms per liter) and *V_i_* is the volume of the correspondent spot urine sample (milliliters). In cases where only one of these two urine samples was collected, the metabolite concentration of the collected sample was used as the DVWA concentration. Urinary concentrations of OP metabolites were not adjusted by creatinine or specific gravity.

## Results

Frequencies of detection (Table 1) for five OP metabolites in 724 urine samples collected from 23 children throughout the study period differed during the conventional diet phases (phases 1 and 3) and varied significantly between conventional and organic diet phases for two metabolites [malathion dicarboxylic acid (MDA), metabolite of malathion; 3,5,6-trichloro-2-pyridinol (TCPY), metabolite of chlorpyrifos]. These differences probably reflect the frequency of the uses of these OP pesticides in agricultural production, in which malathion and chlorpyrifos are all commonly used on fruits, vegetables, and wheat. *para*-Nitrophenol concentration was quantified but not included in this report because it is no longer considered a specific biomarker for methyl parathion exposure (Barr et al. 2002).

**Figure 1:**
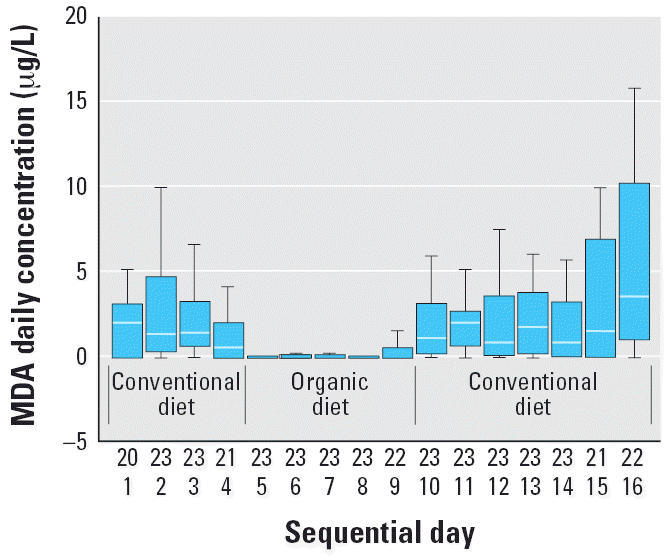
Box plots of DVWA of MDA concentrations in 23 children 3–11 years of age for 15 consecutive days in which conventional and organic diets were consumed. The top row of numbers on the *x*-axis represents numbers of children.

**Figure 2:**
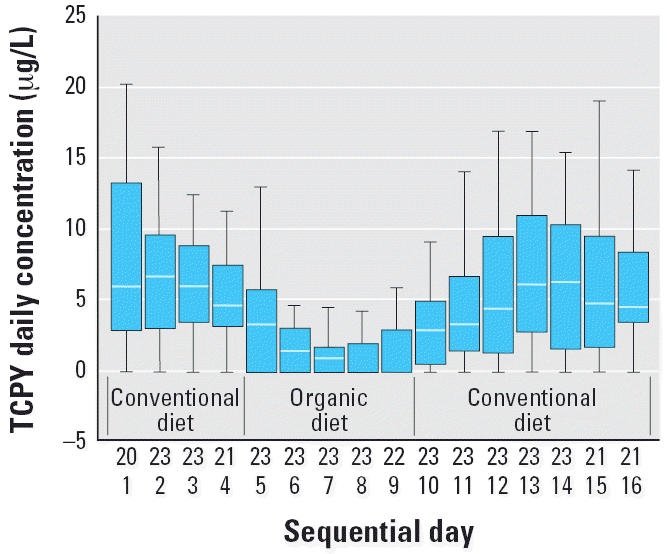
Box plots of DVWA of TCPY concentrations in 23 children 3–11 years of age for 15 consecutive days in which conventional and organic diets were consumed. The top row of numbers on the *x*-axis represents numbers of children.

The distributions of DVWA concentrations for MDA and TCPY during the three study phases (Figures 1 and 2) highlight the effect of organic food consumption on OP pesticide exposures in children. All 23 children’s urine samples contained MDA and TCPY when they enrolled in this study. Immediately after the introduction of organic food to children’s diets, median urinary MDA and TCPY concentrations decreased to the ND level, where they remained until conventional diets were reintroduced after 5 days of organic food consumption. The DVWAs for MDA and TCPY in the organic diet phase were significantly lower than the levels in either conventional diet phase [one-way analysis of variance (ANOVA), *p* < 0.01; Table 1]. The substitution of organic diets had no effect on the dietary exposures for diazinon [parent OP pesticide for 2-isopropyl-6-methyl-pyrimidin-4-ol (IMPY)], methyl pirimiphos [parent OP pesticide for 2-diethylamino-6-methylpyrimidin-4-ol (DEAMPY)], and coumaphos (parent OP pesticide for 3-chloro-4-methyl-7-hydroxycoumarin (CMHC)]. These OP pesticides either are less commonly used in agriculture or have restricted use, such as coumaphos, which is registered for use in livestock only.

## Discussion

Concerns were raised by the NRC (1993) regarding the quantitative and qualitative differences in the toxicity of, and the exposure to, pesticides in children, compared with adults. The NRC report recognized that dietary intake of pesticides represents the major source of exposure for infants and children and concluded that the differences in dietary exposure to pesticide residues account for most of the differences in pesticide-related health risks that were found to exist between children and adults. Dietary pesticide exposure was commonly assessed by collecting duplicate food samples from the study subjects. This method assumes that the pesticide residues measured in the food samples represent the best surrogate measurements for the dietary intake of pesticide residues; however, the correspondence of pesticide residues in the duplicate food samples and the absorbed pesticide dose measured in biologic samples has rarely been determined. The objective of this study was to assess dietary pesticide exposures in individual children by substituting their conventional diets with organic food items. The results from this study should provide the most direct and relevant data for assessing children’s pesticide exposure through dietary intake.

An important aspect of this study was to assure that the study protocol did not alter children’s diets, qualitatively speaking, from their normal consumption patterns. Children may reject organic food items because of taste or appearance and therefore restrict themselves to a rather simple and less diverse diet during the organic diet phase. Consequently, this may confound the results because one cannot determine whether potentially observed differences in dietary pesticide exposures truly result from consuming organic food items and not from changes in children’s diets. We performed a few trial runs with different children in the same age range before the study to evaluate children’s acceptance of organic versions of the food items they regularly ate. Parents commented about their child’s response and found that most items were acceptable to their children, especially children considered not very selective in food choices. We then determined that it would be vital to recruit children who are considered not very selective about food taste and appearance. According to the food diaries, children consumed approximately two more items of fresh produce (including juices) and wheat/rice/soybean-based food items in the organic diet phase, comparing with the conventional diet phase. This finding indicates that the study protocol did not change children’s regular diet consumption pattern and therefore should not bias the study results.

We conclude that organic diets provide a protective mechanism against OP pesticide exposure in young children whose diets regularly consist of fresh fruits and vegetables, fruit juices, and wheat-containing items. Such protection is dramatic and immediate. This is particularly true for certain OP pesticides, such as chlorpyrifos and malathion, as measured in this study, and is probably true for other OP pesticides such as azinphosmethyl, dimethoate, and acephate, which are registered only for agricultural production. These results are consistent with our previous finding (Lu et al. 2001) that none of the dialkylphosphate compounds, a group of nonspecific urinary OP pesticide metabolites, were found in one child from a pool of 110 children. The parents of this child reportedly provided exclusively organic produce and did not use any pesticides at home. Although we did not collect health outcome data in this study, it is intuitive to assume that children whose diets consist of organic food items would have a lower probability of neurologic health risks, a common toxicologic mechanism of the OP pesticide class. The persistent existence of OP pesticide metabolites in urine during the conventional diet periods raises a concern of the possible chronic exposures to OP pesticides in children. However, caution should be exercised when inferring exposures and health risks solely based on OP urinary metabolite levels. Recent studies have suggested that the OP metabolites can occur as degradates either in food commodities (Lu et al. 2005) or in the environment (Morgan et al. 2005), although the amount of metabolites measured represents only a fraction of OP pesticides. The presence of OP pesticide metabolites in foods and in the environment definitely complicates the estimation of absorbed pesticide doses but should not be used to defend a lower likelihood of direct dietary exposure to OP pesticides. If these degradates are absorbed efficiently and excreted unchanged in urine, they could contribute to the total OP metabolite levels. Future researches should be conducted to determine the magnitude of OP pesticide degradation in the environment and in foods, and the pharmacokinetics of those metabolites in humans.

The lack of residential pesticide use as reported by the parents suggests that children in this study were exposed to OP pesticides exclusively from dietary intakes. Recent regulatory changes (U.S. Environmental Protection Agency 1998) aiming to reduce exposures in children have banned or restricted the use of many OP pesticides in the residential environment. This policy change no doubt greatly minimizes the OP pesticide exposures from residential use (Hore et al. 2005; Whyatt et al. 2004); however, fewer restrictions have been imposed in agriculture. Chlorpyrifos and malathion residues in selected food commodities were regularly detected in selected food commodities (Table 2) as surveyed by annual USDA PDP from 2000 to 2003 (USDA 2005). These food items were also commonly consumed by the children in this study. Unfortunately, the trend in agricultural use of these OP pesticides was not assessable after 2002 because completely different commodities were monitored in 2003. The trade-off of the heath risks caused by OP pesticide in children by such regulatory change, therefore, is difficult to quantify.

Last, the magnitude of variability associated with urinary OP pesticide metabolite levels measured in this study is rather large, suggesting that the scenario of dietary exposure is sporadic with significant temporal variations. Such variability reflects the combination of the variation of OP pesticide residues found in food items, the probability of consuming those food items, and the relatively short biologic half-lives of the OP pesticides in humans. The pitfall of such large variability is that it may compromise the true association between exposure and the outcome of interest. Despite this inherent variability, statistically significant trends were evident in this study. A study design that incorporates daily repeatable specimen collection over a period of time in consideration of the pharmacokinetics of the nonpersistent pesticides, such as OP pesticides, is preferable. Spot first morning void urine sample has been suggested as the best representative measurement for the daily OP pesticide exposure (Kissel et al. 2005). However, such an approach is deemed not sufficient for assessing dietary exposure to OP pesticides or other exposure scenarios in which subjects’ activities, such as dietary consumption patterns, are dynamic in nature. Many first morning void urine samples collected in this study had no detectable OP metabolite levels, whereas the before bedtime urine samples collected the previous day contained detectable metabolite levels. Depending upon the timing of pesticide residue intake with certain meals, first morning void urine samples may not represent true exposures. Considering the burden of study subjects and the cost of sample analysis, collecting before bedtime and first morning void samples for assessing dietary exposure to OP pesticides seems to be the best choice.

Children and their families participating in this study do not reflect the general U.S. population, and therefore no attempt should be made to extend this conclusion to other children. It will be of interest, from the regulatory and public health points of view, to conduct additional studies that include children living in homes where residential pesticide use is common. If not applied according to label instructions, pesticide use in or around households may contribute more exposure to residents, particularly children, than does dietary intake (Lu et al. 2001).

## Conclusion

We used a novel study design to provide a convincing demonstration of the ability of organic diets to reduce children’s OP pesticide exposure and the health risks that may be associated with these exposures. This reduction in exposure was dramatic and immediate for the OP pesticides malathion and chlorpyrifos, which are commonly and predominantly used in agricultural production and have no or minimal residential uses. The findings for the OP pesticide exposure in children from this study therefore support the conclusion made by the NRC (1993) that dietary intake of pesticides could represent the major source of exposure in infants and young children.

## Figures and Tables

**Table 1:** Descriptive statistics for the DVWA concentrations of urinary metabolites for selected OP pesticides in the three study phases.

Study phase	No.	Frequency of detection (%)	Median (μg/L)	Mean ± SD (μg/L)	Maximum (μg/L)
MDA (LOD = 0.3 μg/L)					
1	87	60	1.5	2.9 ± 5.0*	96.5
2	116	22	0	0.3 ± 0.9*	7.4
3	156	60	1.6	4.4 ± 12.4*	263.1
TCPY (LOD = 0.2 μg/L)					
1	87	78	6.0	7.2 ± 5.8**	31.1
2	116	50	0.9	1.7 ± 2.7**	17.1
3	155	78	4.3	5.8 ± 5.4**	25.3
IMPY (LOD = 0.7 μg/L)					
1	71	14	0	< LOD ± 0.2	1.2
2	107	9	0	< LOD ± 0.1	0.4
3	148	14	0	< LOD ± 1.3	14.6
DEAMPY (LOD = 0.2 μg/L)					
1	70	25	0	0.37 ± 2.2	17.4
2	103	25	0	< LOD ± 0.1	0.8
3	146	25	0	< LOD ± 0.3	2.3
CMHC (LOD = 0.2 μg/L)					
1	87	25	0	< LOD ± 0.03	0.2
2	115	25	0	< LOD ± 0.03	0.2
3	156	25	0	< LOD ± 0.04	0.2

Abbreviations: CMHC, 3-chloro-4-methyl-7-hydroxycoumarin; DEAMPY, 2-diethylamino-6-methylpyrimidin-4-ol; IMPY, 2-isopropyl-6-methyl-pyrimidin-4-ol; MDA, malathion dicarboxylic acid; TCPY, 3,5,6-trichloro-2-pyridinol.

*Significantly different (one-way ANOVA, *p* < 0.01; Tukey test, phase 2 level significantly lower than levels in phase 1 and 3).

**Significantly different (one-way ANOVA, *p* < 0.001; Tukey test, phase 2 level significantly lower than phase 1 and 3 levels).

**Table 2:** Frequency of detection (%)^a^ of chlorpyrifos and malathion residues in food items, and the frequency of consumption of those food items by children in summer 2003.

	Chlorpyrifos detection				Malathion detection				
Food item	2000	2001	2002	2003	2000	2001	2002	2003	Frequency of consumption by children^b^
Apples	12	8	1		0	0	0		22
Broccoli		2	3			0	< 1		5
Cantaloupe	< 1			1	0			0	12
Carrot	0	2	7		0	< 1	0		14
Celery		1	3			20	26		2
Cherry	3	1			16	11			8
Grape (and juice)	9	6			< 1	0			15
Nectarine	6	2			< 1	0			4
Orange	1	2			0				5
Peach	30	34	35		0	< 1	< 1		6
Rice	< 1	< 1	< 1		17	11	4		15
Strawberry (fresh)	< 1				18				8
Sweet bell pepper	15		5	18	2		0	1	3
Tomato (canned)^c^	0	9		4	0	0		0	26
Wheat/barley/soybean^d^			4	16			2	38	229

a Data from USDA (2005). Blank cells represent items that were not analyzed for chlorpyrifos or malathion by USDA.

b Total consumption (servings) for 23 children in 15 consecutive days.

c Consumed along with other food, such as pizza, pasta, and spaghetti.

d Including pizza, bagel, bread, cereal, cookies, chips, crackers, and noodles.
